# Genetic response and metabolic adaptation to partial-record selection for egg production in Dokki-4 laying hens

**DOI:** 10.1038/s41598-025-30146-7

**Published:** 2025-12-09

**Authors:** Doaa A.M. Semida, Ahmed M. Emam, Bothaina Y.F. Mahmoud, Ensaf A. ElFull, Mohamed. S. Shalaby, Galal Abou Khadiga

**Affiliations:** 1 Poultry Production Department, Faculty of Agriculture, Fayoum University, Fayoum, 63514 Egypt; 2 Seds Station for Animal Breeding, Animal Production Research Institute, Agriculture Research Center, Ministry of Agriculture, Dokki, Egypt; 3 Faculty of Desert and Environmental Agriculture, Matrouh University, Fuka, Matrouh, 51744 Egypt

**Keywords:** Selection, Egg production, Egg quality, Genetic gain, Serum parameters, Heritability, Correlation, Biochemistry, Genetics, Physiology

## Abstract

This study aimed to evaluate the impact of partial-record egg production selection on egg production performance, serum biochemical constituents, and egg quality traits in Dokki-4 laying hens. A selection experiment was conducted over four generations involving 2,880 hens distributed across three experimental lines: high egg number line (SHEN₉₀), heavy egg weight line (SHEW₉₀), and a random-bred control line (RBCL). Selection criteria were based on performance during the first 90 days of production. Line effects were highly significant for all egg production traits, serum calcium concentrations, yolk cholesterol levels, and most egg quality traits. The SHEN₉₀ line showed superior egg number, annual egg mass production performance, and exhibited favorable metabolic profiles with reduced serum cholesterol and triglycerides. The SHEW₉₀ line achieved significantly heavier egg weights and improved shell quality traits compared to other lines. Genetic gain estimates revealed significant improvements in economically important traits. The SHEN₉₀ line showed favorable genetic gains for EN₉₀, EM₉₀, EM₃₆₅, and favorable reductions in serum cholesterol, triglycerides, and yolk cholesterol. The SHEW₉₀ line exhibited significant positive genetic gains in EW₉₀, EM₉₀, EM₃₆₅, and favorable yolk weight and yolk index percentages. Heritability estimates were moderate for selection criteria (EN₉₀: 0.22; EW₉₀: 0.17) and ranged from 0.09 to 0.31 for correlated traits. Genetic correlations revealed strong positive relationships between EN₉₀ and egg mass traits. EW₉₀ showed positive correlations with most external quality parameters. Selection for partial-record egg production represents an effective breeding strategy for simultaneously improving multiple economically important traits in Dokki-4 laying hens.

## Introduction

The global demand for high-quality eggs is steadily increasing, driven by population growth and a rising recognition of eggs as an affordable, nutrient-dense protein source^1^. This increasing demand has intensified the need for efficient poultry breeding programs that can simultaneously enhance productive performance while maintaining superior egg quality and optimal metabolic health in laying hens. Modern poultry breeding is thus challenged by the need to balance multiple, often competing objectives, such as maximizing egg production, optimizing egg weight, ensuring excellent egg quality, and safeguarding bird welfare through favorable physiological profiles^2^.

In this regard, indigenous chicken breeds play a vital role in addressing these challenges, particularly in developing countries. Their natural adaptation to harsh climates positions them as valuable genetic resources in breeding strategies^3^. Moreover, these native breeds represent important genetic resources that can enrich the genetic diversity of commercial chicken lines by capturing useful genetic variants that exist exclusively in indigenous populations^4^. In Egypt, the Dokki-4 strain represents one of the most prominent local synthetic lines. It was originally developed by crossbreeding native Fayoumi chickens with foreign breeds to combine adaptability with improved egg production traits. Over time, Dokki-4 has become the genetic basis for many local egg-producing strains and remains an important reservoir of genetic diversity in national poultry improvement programs^5^.

When working with such genetic diversity, selection for egg production becomes particularly valuable when multiple economic traits must be simultaneously considered and genetically evaluated for improving overall productivity in local chicken populations^6^. Traditional selection approaches in laying hens typically focus on full-cycle egg production traits, which require prolonged evaluation periods and high maintenance costs. However, there are partial-record selection strategies, particularly those targeting early production periods. This approach enables early identification of superior individuals, reduces generation intervals, and accelerates genetic gain, reducing costs associated with maintaining large breeding populations over extended periods^7^. Notably, partial-record selection, focusing on early production traits such as egg number, has demonstrated improvements in egg quality characteristics^8,9^.

The physiological complexity underlying egg production extends far beyond simple quantitative measures, encompassing intricate metabolic processes involving lipid metabolism, calcium homeostasis, and cholesterol regulation. Genetic selection can significantly alter physiological relationships and metabolic processes, directly or indirectly, affecting egg quality^8,9^, egg production^10^, and growth performance^11^. Understanding how genetic selection for production traits influences these underlying physiological mechanisms is crucial for developing sustainable breeding programs that enhance productivity without compromising bird health or welfare. Thus, a critical knowledge gap remains regarding how partial-record selection for egg number and weight affects not only production performance but also egg quality and serum biochemical profiles in genetically distinct local chicken populations. Yet few studies have systematically evaluated these relationships in local Egyptian strains such as Dokki-4.

This study aimed to evaluate the impact of partial-record selection for early egg number and egg weight on egg production traits, egg quality characteristics (external and internal), and key blood biochemical constituents (serum calcium, triglycerides, and cholesterol) in Dokki-4 laying hens. Additionally, the study estimates genetic parameters including heritability and genetic correlations, offering valuable information for optimizing future breeding strategies in this important local strain.

## Materials and methods

### Experimental design and bird management

This experiment was carried out at Seds Station for Animal Breeding, Animal Production Research Institute, Agriculture Research Center, Ministry of Agriculture, Egypt. This experiment was carried out in compliance with Fayoum University guidelines. The study was approved by Fayoum University Institutional Animal Care and Use Committee (IACUC) under protocol No AFC 2358. The study is reported in accordance with ARRIVE guidelines.

A selection experiment was conducted over four generations using 2880 Dokki-4 hens (a locally developed strain). Three experimental lines were maintained: two selected lines for high egg number (SHEN_90_) and heavy egg weight (SHEW_90_) during the first 90 days of production, and a random-bred control line (RBCL), which was maintained as a non-selected, pedigreed population over generations. Each generation included 240 females linked to 120 males per line. Selection intensities were approximately 15% for females and 10% for males in each generation, applied to the top-ranked birds based on EBVs for EN₉₀ and EW₉₀. Full- and half-sib matings were strictly avoided in all lines to maintain a low inbreeding coefficient. Data completeness and mortality were carefully monitored across all generations. The overall mortality rate from hatch to the end of the laying period averaged less than 5% per generation, with no significant differences among lines.

All birds were transferred to individual laying cages (25 × 35 × 40 cm) at 18 weeks of age. The pullets were fed 120 g/day of a laying diet containing 16% crude protein, 11.72 MJ metabolizable energy, 2.5% calcium, 0.65% phosphorus, 0.85% lysine, and 0.38% methionine throughout the production period. Environmental conditions in the laying house were maintained at 22 ± 2 °C, 60–65% relative humidity. During the production phase, the lighting program was set at 16 h of light and 8 h of dark. Birds were vaccinated against Newcastle disease, infectious bronchitis, and avian influenza according to standard protocols prevalent in the study area.

### Studied traits

Selection criteria were individually recorded for each female included in the experiment. The first trait considered was the total number of eggs laid by each hen during the first 90 days of production (EN_90_). The second trait was the average egg weight over the same 90-day period (EW_90_), which was measured daily for each individual using a high-precision electronic balance scale with a sensitivity of 0.01 g. Another set of traits included egg production-related traits, which were age at first egg laid (AFE), egg mass during the first 90 days of lay (EM_90_), and egg mass during 364 days of age (full record, EM_365_).

### Blood biochemical analysis

Four-hundred and fifty blood samples were collected at 32 weeks of age, assigned from the three lines (150 samples per generation, 50 blood samples for each line). Five ml blood samples were individually taken from the wing vein. The collected samples were centrifuged for 10 min at 4500 rpm and kept at −20 °C until processing. Serum biochemical parameters, including total cholesterol, triglycerides, and calcium concentrations, were assayed on a spectrophotometer using Spain commercial kits (SPINREACT) according to the manufacturer’s recommendations.

### Egg quality traits

Four-hundred and fifty eggs were collected at 32 weeks of age, assigned from the three lines (150 eggs per generation, 50 eggs for each line). Egg quality characteristics, either external (relative weights of shell and shell thickness) or internal (yolk, albumen, yolk index, Haugh units) traits, were calculated^12^ and were examined at 32 weeks of age. Yolk cholesterol was also determined according to^13^.

### Statistical analysis

Preliminary bivariate analyses were conducted, including permanent environmental and maternal effects, but their variance components were below 3%. The result of comparing a model including these effects with the simpler additive animal model using the Likelihood Ratio Test (LRT) was insignificant; therefore, the simpler model was used. Missing records due to mortality or technical loss were handled using the REML procedure in WOMBAT, which incorporates all available pedigree relationships to recover information and minimize bias^14^. Consequently, the final dataset retained full representativeness of the original populations and ensured unbiased estimation of variance components.

The recorded data of the traits studied were analyzed by PROC MIXED^15^ to calculate the generation and line-specific means for all traits using the following model:

$${\text{Y}_\text{ijkl}} = {\text{ }}\mu {\text{ }} + {\text{ }}{\text{a}_\text{i}} + {\text{ }}{\text{G}_\text{j}} + {\text{ }}{\text{L}_\text{k}} + {\text{ }}{\text{G}_\text{j}} \times {\text{ }}{\text{L}_\text{k}} + {\text{ }}{\text{e}_\text{ijkl}}$$

where, Y_ijkl_ is the observation for a trait, µ is the overall mean; a is the random additive genetic effect of the i^th^ hens within line, G is the fixed effect of j^th^ generation, L is the fixed effect of k^th^ line, G_j_ × L_k_ is the interaction of G_j_ and L_k_ and e_ijkl_ is the random error term. Means of generations and their interactions with the lines were compared using multiple range test^16^. A significance level (α) of 0.05 was used to determine statistical significance.

### Genetic parameter Estimation

Genetic parameters for all studied traits were estimated using variance components derived from REML analysis^14^, with a convergence criterion set at 10⁻⁸ for all analytical runs through linear animal models as univariate for estimating heritabilities (h²) of studied traits and bivariate for calculating correlations according to REML procedures by WOMBAT software.

The univariate model to estimate direct h² was:

$$\text{y}{\text{ }} = {\text{ }}\text{Xb}{\text{ }} + {\text{ }}\text{Za}{\text{ }} + {\text{ }}\text{e}$$

The bivariate model to estimate correlations between the selection criterion trait and other studied traits was:

$$\:\left[\begin{array}{c}{\text{y}}_{1}\\\:{\text{y}}_{2}\end{array}\right]=\:\left[\begin{array}{cc}{\text{x}}_{1}&\:0\\\:0&\:{\text{x}}_{2}\end{array}\right]\left[\begin{array}{c}{\text{b}}_{1}\\\:{\text{b}}_{2}\end{array}\right]\:+\:\left[\begin{array}{cc}{\text{z}}_{1}&\:0\\\:0&\:{\text{z}}_{2}\end{array}\right]\left[\begin{array}{c}{\text{a}}_{1}\\\:{\text{a}}_{2}\end{array}\right]+\:\left[\begin{array}{c}{\text{e}}_{1}\\\:{\text{e}}_{2}\end{array}\right]$$

where: for trait i (i = 1, 2), y_i_=vector of observations, b_i_= vector of fixed effects (i.e., generation and line), a_i_=vector of random direct genetic effects, e_i_=vector of random residual effects, and X_i_ and Z_i_ are incidence matrices relating the observations to the respective fixed and direct genetic effects.

Starting values for these calculations were obtained through preliminary multivariate REML analyses incorporating complete pedigree information of the population. Genetic gain was evaluated using estimated breeding values (EBV) calculated through mixed model methodology^14^. Genetic gain per generation was quantified as the difference between the mean EBV of the fourth and first generations within each selected line.

## Results

### Egg production traits

In the present study, significant generation effects were observed for the selection criteria (EN_90_ and EW_90_) and for EM_90_ (Table 1). Both EN_90_ and EM_90_ means exhibited a decreasing trend across generations, yet with significant differences. Meanwhile, EW_90_ means varied significantly among generations, though without a consistent directional pattern. However, no significant generation effects were detected for AFE, EN_365,_ and EM_365_. The line effect was highly significant (*P* < 0.001) for all egg production traits. The SHEN_90_ line outperformed the other lines in EN_90_, EN_365_, and both egg mass traits (EM_90_ and EM_365_), while the SHEW_90_ line produced significantly heavier eggs (EW_90_) than other lines. Furthermore, the generation x line interaction had a significant influence on both EN_90_ and EM_90,_ indicating differential responses to selection across generations among the three genetic lines.

**Table 1:** Least squares means ± SE for selection criteria and other studied egg production traits as affected by generation and line effects.

		Selection criteria		Egg production traits			
		EN_90_ (egg)	EW_90_ (g)	AFE (days)	EM_90_(g)	EM_365_ (g)	EN365
Generation	2	38.63 ± 1.43^a^	39.96 ± 0.53^ab^	179.65 ± 3.05	1525.0 ± 69.09^a^	4930.4 ± 112.94	113.53 ± 3.52
	3	30.47 ± 1.03^b^	40.66 ± 0.38^a^	173.17 ± 2.19	1214.5 ± 56.83^b^	5118.6 ± 115.63	112.37 ± 2.53
	4	29.19 ± 1.40^b^	38.94 ± 0.52^b^	182.86 ± 2.99	1177.5 ± 64.23^c^	4939.4 ± 110.24	111.65 ± 3.45
Line	SHEN_90_	58.05 ± 1.32^a^	38.22 ± 0.49^b^	161.60 ± 2.81^c^	1828.81 ± 70.24^a^	5969.5 ± 114.32^a^	141.97 ± 3.26^a^
	SHEW_90_	26.20 ± 1.25^b^	42.29 ± 0.46^a^	183.24 ± 2.67^b^	1094.10 ± 69.40^b^	4776.3 ± 114.56^b^	100.07 ± 3.09^b^
	RBCL	21.97 ± 1.32^c^	39.26 ± 0.49^b^	190.56 ± 2.81^a^	901.74 ± 71.24^c^	4290.0 ± 122.32^c^	95.51 ± 3.25^b^
*P-*value	Generation	0.001	0.026	0.052	0.002	0.502	0.092
	Line	0.001	0.001	0.001	0.001	0.001	0.0001
	Generation X Line	0.001	0.312	0.059	0.008	0.464	0.153

AFE: Age at the first laid egg (days); EN_90_: The total number of eggs laid by individual layer till 90 days of production; EW_90_: average egg weight during 90 days of lay; EM_90_: Egg mass during the first 90 days of lay; EM_365_: Egg mass during 365 days of age; SHEN_90_: The selected line for higher egg number during first 90 days of production; SHEW_90_: The selected line for heavier egg weight during first 90 days of production; SEM: Standard error of mean.

Means in the same column with different superscript letters (^a, b,c^) are significantly different (*P* < 0.05).

### Serum constituents

Significant generation effects were observed for serum calcium and triglycerides (*P* < 0.001), whereas total serum cholesterol was not significantly affected (*P* = 0.192). Across the three generations, triglycerides and total cholesterol levels exhibited a decreasing trend, while serum calcium concentrations showed a consistent increase (Table 2). Regarding line effects, serum calcium was significantly influenced (*P* < 0.001), with the SHEN_90_ line displaying the highest calcium levels, significantly surpassing those of other lines. However, line differences in triglycerides and cholesterol were not statistically significant. A significant generation × line interaction was detected for both serum calcium and total serum cholesterol (*P* = 0.023 and 0.001, respectively), suggesting that the metabolic response to selection varied across genetic lines over generations. No significant interaction was found for serum triglycerides (*P* = 0.451).

**Table 2:** Least squares means ± SE for the serum calcium, serum triglycerides, and total cholesterol as affected by generation and line effects.

		Serum Calcium (mg/dl)	Serum Triglycerides (mg/dl)	Total Serum Cholesterol (mg/dl)
Generation	2	19.68 ± 0.10^c^	548.50 ± 9.94^a^	132.46 ± 3.14
	3	22.36 ± 0.11^b^	424.63 ± 10.83^b^	126.78 ± 3.31
	4	24.69 ± 0.10^a^	412.42 ± 10.68^b^	124.69 ± 3.08
Line	SHEN₉₀	23.91 ± 0.11^a^	469.11 ± 10.13	128.78 ± 3.24
	SHEW₉₀	20.25 ± 0.10^b^	474.26 ± 10.68	130.90 ± 3.08
	RBCL	22.67 ± 0.11^a^	447.17 ± 10.04	124.09 ± 3.21
*P*-value	Generation	0.001	0.001	0.192
	Line	0.001	0.076	0.295
	Generation × Line	0.023	0.451	0.001

SHEN₉₀: Selected line for higher egg number during the first 90 days of production; SHEW₉₀: Selected line for heavier egg weight during the first 90 days of production.

Means in the same column with different superscript letters (^a, b,c^) are significantly different (*P* < 0.05).

### External egg quality traits

External egg quality traits are presented in Table 3. Shell weight% and shell thickness were significantly influenced by generation (*P* = 0.001 and 0.049, respectively), with both parameters showing a decreasing trend across generations, while the shape index was not significantly influenced (*P* = 0.173). The second generation exhibited superior shell weight% and shell thickness compared to other generations. Regarding line effects, shell thickness differed significantly among lines (*P* = 0.001), with the SHEW₉₀ line showing the greatest shell thickness. However, no significant differences were found among lines for shell weight% or shape index. The generation × line interaction had a significant effect on shell thickness (*P* = 0.029), suggesting that the impact of selection on this trait varied depending on the combination of generation and genetic line. No significant interactions were observed for shell weight% or shape index.

**Table 3:** Least squares means ± SE for the studied external egg quality traits as affected by generation and line effects.

		Shell weight%	Shell thickness, mm	Shape index
Generation	2	13.37 ± 0.22^a^	0.42 ± 0.004^a^	78.35 ± 0.55
	3	13.01 ± 0.16^a^	0.41 ± 0.003^ab^	79.18 ± 0.40
	4	12.01 ± 0.21^b^	0.40 ± 0.004^b^	79.18 ± 0.55
Line	SHEN_90_	12.32 ± 0.20	0.40 ± 0.004^b^	78.75 ± 0.51
	SHEW_90_	12.73 ± 0.19	0.42 ± 0.003^a^	78.88 ± 0.50
	RBCL	12.71 ± 0.20	0.40 ± 0.004^b^	79.35 ± 0.51
*P-*value	Generation	0.001	0.049	0.173
	Line	0.196	0.001	0.419
	Generation X Line	0.515	0.029	0.150

SHEN_90_: The selected line for higher egg number during the first 90 days of production; SHEW_90_: The selected line for heavier egg weight during first 90 days of production; SE: Standard error.

Means in the same column with different superscript letters (^a, b^) are significantly different (*P* < 0.05).

### Internal egg quality traits

Table 4 summarizes the internal egg quality traits and yolk cholesterol content as affected by generation and genetic line. All of the internal egg quality traits (yolk weight%, Haugh units, yolk index, and yolk cholesterol) were significantly affected by generation, while albumen weight%, which mostly showed constant estimates, remained unaffected (*P* = 0.808). A significantly desirable increase in yolk weight% was found across generations, increasing from 31.09% in the second generation to 32.62% in the fourth generation. In contrast, Haugh units decreased from generation 2 to generation 3, then partially recovered in generation 4. Yolk cholesterol showed a similar trend with more marked reduction between generation 2 and later generations, reflecting potential improvements in lipid metabolism. Another fluctuating trend was observed for the yolk index, which increased from generation 2 to generation 3, then dropped to its lowest level in generation 4.

**Table 4:** Least squares means ± SE for the studied internal egg quality traits and yolk cholesterol as affected by generation and line effects.

		Albumen weight%	Yolk weight%	Haugh units	Yolk index	Yolk cholesterol, mg/g
Generation	2	55.54 ± 0.61	31.09 ± 0.26^b^	80.35 ± 0.90^a^	43.20 ± 0.61^ab^	12.1 ± 0.30^a^
	3	55.89 ± 0.44	31.62 ± 0.18^b^	72.22 ± 0.65^c^	44.23 ± 0.44^a^	9.68 ± 0.22^b^
	4	55.46 ± 0.61	32.62 ± 0.26^a^	75.21 ± 0.89^b^	43.07 ± 0.61^b^	10.35 ± 0.27^b^
Line	SHEN_90_	55.62 ± 0.57	32.06 ± 0.24^a^	75.07 ± 0.84	45.15 ± 0.57^a^	10.09 ± 0.28^b^
	SHEW_90_	56.15 ± 0.55	31.12 ± 0.23^b^	74.63 ± 0.79	42.41 ± 0.55^c^	10.12 ± 0.27^b^
	RBCL	55.22 ± 0.56	32.06 ± 0.24^a^	75.40 ± 0.84	43.63 ± 0.57^b^	11.19 ± 0.28^a^
*P-*value	Generation	0.808	0.001	0.001	0.037	0.001
	Line	0.463	0.002	0.777	0.001	0.005
	Generation X Line	0.043	0.001	0.001	0.001	0.038

SHEN_90_: The selected line for higher egg number during the first 90 days of production; SHEW_90_: The selected line for heavier egg weight during the first 90 days of production; SE: Standard error.

Means in the same column with different superscript letters (^a, b,c^) are significantly different (*P* < 0.05).

Regarding line effects, significant differences were observed in all yolk characters (yolk weight%, yolk index, and yolk cholesterol). Both SHEN₉₀ and RBCL had identical higher yolk weight percentages (32.06%) compared to SHEW₉₀. The SHEN₉₀ line showed the highest yolk index (45.15%) and the lowest yolk cholesterol. The SHEW₉₀ line had the lowest yolk weight and yolk index, yet yolk cholesterol values were close to SHEN₉₀. Yolk cholesterol was significantly affected by line, favoring the two selected lines, which had lower estimates (10.09 and 10.12 mg/g for SHEN₉₀ and SHEW₉₀, respectively) compared to the RBCL line. Albumen weight and Haugh units were not significantly affected by line.

Importantly, generation × line interactions were significant for all traits, indicating that the selected lines responded differently across generations for these traits.

### Genetic gain and correlated changes

The estimates of heritability and genetic gain for various traits in the SHEN₉₀ and SHEW₉₀, along with their heritability estimates, are presented in Table 5.

**Table 5:** Heritability estimates ± standard error (h^2^ ± SE) and genetic gain of the studied traits in the selected lines.

	h^2^ ± SE	SHEN_90_	SHEW_90_
**Selection criteria**			
EN_90_	0.22 ± 0.08	9.49 ± 0.31*	−1.41 ± 0.22
EW_90_	0.17 ± 0.07	−2.23 ± 0.16	2.39 ± 0.19*
Egg production traits			
AFE, days	0.09 ± 0.06	−0.31 ± 0.04	0.88 ± 0.07
EM_90_, g	0.21 ± 0.08	82.38 ± 8.11*	51.80 ± 4.77*
EM_365_, g	0.15 ± 0.07	76.62 ± 6.91*	40.64 ± 4.88*
EN_365, egg_	0.21 ± 0.001	15.2 ± 0.58*	−2.1 ± 0.35
**Serum constituents traits**			
Cholesterol, mg/dl	0.31 ± 0.06	−4.57 ± 0.39*	2.65 ± 0.22
Triglycerides, mg/dl	0.23 ± 0.07	−45.95 ± 4.12*	−48.37 ± 4.90*
Calcium, mg/dl	0.21 ± 0.05	0.53 ± 0.03	0.25 ± 0.03
**Egg quality traits**			
Shell weight%	0.19 ± 0.021	−0.13 ± 0.03	0.78 ± 0.04
Shell thickness, mm	0.16 ± 0.031	0.05 ± 0.01	0.04 ± 0.01
Shape index %	0.17 ± 0.011	−0.31 ± 0.01	0.28 ± 0.01
Albumen weight%	0.09 ± 0.006	−1.39 ± 0.2	0.37 ± 0.01*
Yolk weight%	0.11 ± 0.012	−0.62 ± 0.01	1.07 ± 0.03
Yolk index %	0.11 ± 0.036	−0.43 ± 0.01	0.95 ± 0.02
Yolk cholesterol, mg/g	0.16 ± 0.070	−5.32 ± 0.48*	2.44 ± 0.31

SHEN_90_: The selected line for higher egg number during first 90 days of production; SHEW_90_: The selected line for heavier egg weight during first 90 days of production; EN_90_: Average egg number during 90 days of lay EW_90_: Average egg weight during 90 days of lay, AFE: Age at first laid egg (days), EM_90_: Egg mass during the first 90 days of lay (g); EM_365_: Egg mass during 365 days of age (g); SE: Standard error.

*: (*P* < 0.05).

The present study revealed moderate h² estimates for both selection criteria (EN_90_: 0.22 and EW_90_: 0.17). Similar moderate h² values, ranging from 0.15 to 0.21, were found for egg production traits, metabolic parameters (serum calcium, triglycerides, total cholesterol), shell weight%, shape index, and yolk cholesterol. In contrast, AFE, shell thickness, albumen weight%, yolk weight%, and yolk index demonstrated low heritability estimates ranging from 0.09 to 0.11 (Table 5). These results indicate that most traits responded moderately to selection, with stronger genetic control observed for blood lipids and egg mass traits.

In the SHEN_90_ line, significant favorable genetic gains (*P* < 0.05) were observed for EN_90_, confirming the effectiveness of selection for early egg number. A similar trend was observed for EM_90_, EM_365_, serum cholesterol, serum triglycerides, and yolk cholesterol. However, no significant changes were detected for serum calcium or albumen weight. A mild improvement in age at first egg (AFE) was observed in the SHEN₉₀ line.

The SHEW_90_ line exhibited significant positive genetic gains (*P* < 0.05) for EW_90_, EM_90_, EM_365_, and albumen weight%. Additionally, a significant desirable decrease in serum triglycerides and an increase in albumen weight% were observed. Although favorable, genetic gain estimates for external eggshell traits and internal egg quality parameters were not statistically significant. Unfavorable but insignificant increases were noted for AFE, serum cholesterol, and yolk cholesterol, along with a slight increase in serum calcium.

Overall, when comparing both lines, genetic responses are expected in the selection criteria and the correlated traits. For serum biochemical traits, both lines showed marked reductions in triglyceride levels, and the SHEN₉₀ line exhibited a significant decrease in cholesterol concentration (− 4.57 mg/dl; *P* < 0.05), suggesting favorable metabolic adaptations to selection. In terms of egg quality traits, the SHEW₉₀ line achieved greater improvements, particularly in albumen weight (+ 0.37%; *P* < 0.05), yolk weight, and yolk index, consistent with its selection direction. However, the SHEN₉₀ line showed a greater reduction in yolk cholesterol (− 5.32 mg/g; *P* < 0.05), which may be interpreted as a favorable response from a nutritional standpoint. The genetic responses tended to be opposite in direction, with negative estimates observed in the SHEN₉₀ line and positive estimates in the SHEW₉₀ line. However, most of these responses were not statistically significant, indicating limited selection effects on these traits.

### Genetic and phenotypic correlations

Table 6 shows the genetic (r₉) and phenotypic (rₚ) correlations between the selection criteria (EN₉₀ and EW₉₀) and a range of productive, physiological, and egg quality traits. The correlations revealed important relationships that explain the selection responses observed in both SHEN₉₀ and SHEW₉₀ lines.

**Table 6:** Genetic (r_g_) and phenotypic (r_p_) correlations among the selected and correlated traits and their standard errors (SE).

Traits	EN_90_ (egg)		EW_90_ (g)	
	*r*_g_±SE	*r*_*p*_± SE	*r*_g_± SE	*r*_*p*_± SE
AFE	−0.61 ± 0.07	−0.75 ± 0.06	0.24 ± 0.06	0.13 ± 0.02
EM90	0.71 ± 0.02	0.78 ± 0.11	0.30 ± 0.03	0.15 ± 0.03
EM365	0.66 ± 0.09	0.74 ± 0.09	0.21 ± 0.02	0.07 ± 0.02
EN365	0.78 ± 0.06	0.56 ± 0.09	−0.19 ± 0.04	−0.04 ± 0.01
Serum cholesterol	0.27 ± 0.08	0.14 ± 0.05	0.12 ± 0.10	0.09 ± 0.04
Serum triglycerides	0.46 ± 0.08	0.19 ± 0.03	−0.05 ± 0.02	−0.10 ± 0.04
Serum calcium	0.18 ± 0.06	0.14 ± 0.03	0.35 ± 0.13	0.20 ± 0.06
Yolk cholesterol	0.08 ± 0.01	0.02 ± 0.01	0.22 ± 0.12	0.04 ± 0.01
Shape Index	−0.07 ± 0.01	−0.01 ± 0.01	0.16 ± 0.04	0.06 ± 0.01
Shell Thickness	−0.12 ± 0.01	−0.08 ± 0.01	0.07 ± 0.04	0.04 ± 0.01
Yolk Index	−0.05 ± 0.01	−0.01 ± 0.01	−0.03 ± 0.02	−0.01 ± 0.01
Shell Weight%	−0.15 ± 0.01	−0.04 ± 0.01	0.07 ± 0.02	0.09 ± 0.01
Albumin Weight%	0.05 ± 0.01	0.02 ± 0.01	0.11 ± 0.03	0.04 ± 0.01
Yolk weight%	−0.19 ± 0.04	−0.07 ± 0.01	0.08 ± 0.02	0.03 ± 0.01

SHEN_90_: The selected line for higher egg number during first 90 days of production; SHEW_90_: The selected line for heavier egg weight during first 90 days of production; EN_90_: Average egg number during 90 days of lay EW_90_: Average egg weight during 90 days of lay, AFE: Age at first laid egg (days), EM_90_: Egg mass during the first 90 days of lay (g); EM_365_: Egg mass during 36*5* days of age (g); SE: Standard error.

Strong, favorable genetic (−0.61) and phenotypic (−0.75) correlations were observed between EN₉₀ and AFE, indicating that selection for higher early egg number is associated with earlier sexual maturity. Contrarily, EN₉₀ showed strong positive genetic correlations with egg mass traits, including EM₉₀ (0.71) and EM₃₆₅ (0.66), with corresponding phenotypic correlations of 0.78 and 0.74, respectively. A strong positive genetic correlation was also found between EN₉₀ and EN₃₆₅ (0.78), supporting the effectiveness of partial-record selection, confirming that early production is a reliable predictor of long-term productivity. Correlations between the selection criterion (EN₉₀) and serum biochemical traits were generally low to moderate. Moderate genetic correlations were found with serum triglycerides (0.46) and cholesterol (0.27), indicating that selection for early egg number may be associated with altered lipid metabolism. Phenotypic correlations were not that far from genetic correlation values except for serum triglycerides (0.19). Regarding egg quality traits, EN₉₀ tended to have low negative genetic and phenotypic correlations with most quality parameters, except for a low positive correlation with Yolk cholesterol and albumen weight%.

EW₉₀ showed moderate positive genetic correlations with productive traits (AFE, EM₉₀, and EM_365_), while negative correlations were observed with EN₃₆₅, confirming the antagonistic relationship between egg weight and egg number. A similar trend for phenotypic correlations of these traits was observed with weaker values. For serum constituents, EW₉₀ exhibited low positive genetic correlations with serum calcium and cholesterol (0.35 and 0.22, respectively), while showing negative low correlations with serum triglycerides. EW₉₀ showed slightly positive correlations with shell quality traits, and yolk weight, particularly shell weight, albumen weight, and shell thickness.

None of the correlations between selection criteria, on one side, and egg quality traits from the other side exceeded moderate levels, suggesting limited indirect responses in external or internal egg quality when selecting for EN₉₀ or EW₉₀.

Overall, the correlation patterns indicate that EN₉₀ is a stronger selection driver for long-term productivity, while EW₉₀ is more weakly associated with physiological and quality traits, reinforcing the importance of multi-trait selection strategies.

## Discussion

In the current study, a local laying strain (Dokki-4) was used to assess the effects of partial-record selection on early egg production traits and their correlated responses over several generations. The findings highlighted the biological and genetic effects of the selection strategy by showing notable changes in serum biochemistry, moderate shifts in egg quality traits, and significant genetic gains in selection criteria. In the current study, a local laying strain (Dokki-4) was used to assess the effects of partial-record selection. The observed results align with^8^, though mean EN₉₀ values were lower in our control line, likely due to management differences and genetic drift in the unselected control population.

### Egg production traits

The selection responses in egg production traits provided clear evidence of the effectiveness of early-record-based selection strategies. Significant generation effects were observed for EN₉₀, EW₉₀, and EM₉₀ (Table 1), confirming that selection based on early production records successfully altered performance.

In the current study, the EN_90_ of the SHEN_90_ line exceeded those reported by^9^, and outperformed Horro chickens^17^ and Golden Sabahia^18^ but was lower than the values reported by^8^. This variation may be attributed to differences in flock structure, selection intensity, selection criteria, number of selected generations, and genetic background among the studied strains. The decrease in EN_90_ means across selection generations could be explained by the poor egg production performance of the RBCL line in the third and fourth generations, which likely influenced the EN_90_ means of these generations. It could also be attributed to management differences and genetic drift in the unselected control population.

Interestingly, the EN₃₆₅ trait showed only moderate sensitivity to selection, and no significant generation effect was detected, suggesting that early egg number is a better predictor of short-term productivity than of full-cycle laying performance. The observed results align with^8^ who reported heavier EW_90_ averages than those observed in the current study, while^6,9^ reported lower EW_90_ values than our findings. The age at first egg in our study was comparable to that reported by^17^ for improved Horro chickens, indicating similar sexual maturity patterns in local breeds. This consistency with some studies while differing from others reflects the complex interplay between genetic selection and physiological adaptation mechanisms in laying hens^19^. Significant effects of generation, line, and generation × line interaction on EN_90_, EW_90_, AFE, and EM_90_ in different chicken strains were found by^8,9^. The observed means of EM_90_ and EM_365_ were lower than those reported by^8^. However, the current results of EM_90_ were similar to those of^9^ and higher than those reported by^18^ in Golden Sabahi chicken. This consistency with some studies, while differing from others, reflects the complex interaction between genetic potential, environmental management, and selection intensity. It also underscores that our control group was deliberately maintained to preserve genetic contrast, which explains the lower EN₉₀ means compared with earlier reports.

### Serum constituents

Selection also triggered notable physiological adaptations, particularly in lipid metabolism and calcium homeostasis, as reflected in changes in serum constituents (Table 2). The metabolic responses to selection for egg production traits revealed complex interactions between lipid metabolism, calcium homeostasis, and cholesterol regulation^20^. Serum calcium levels showed a significant generation effect, with values aligning with ranges reported in laying hens under different dietary conditions^20–23^. The parallel increase in serum calcium with egg production levels suggests successful metabolic adaptation, potentially mediated by gonadotropin hormones, which play a crucial role in regulating blood calcium levels in laying hens^24^. Although serum calcium increased across generations, the differences among lines were modest. In the current study, the SHEN_90_ line’s superior egg production associated with higher calcium levels suggests that elevated calcium concentrations both facilitate and respond to increased egg production, indicating efficient calcium cycling.

The serum lipid profile revealed significant patterns in both triglycerides and cholesterol metabolism across generations and lines. Triglyceride levels aligned closely with those reported by^25^ in their negative control group. However, the values were lower than those reported by^26^. Regarding cholesterol levels, the findings of this study were higher than the values observed by both^25,26^. The maintenance of moderate cholesterol levels suggests an optimal balance between lipid metabolism and egg production requirements, as supported by^27^. Birds with more efficient lipid metabolism are capable of maintaining adequate substrate availability for egg production while avoiding excessive lipid accumulation in the bloodstream^28^. The decreasing trend of serum triglycerides and cholesterol across generations suggests that selection for early partial-record production, particularly for EN₉₀, may improve lipid metabolism, a potentially favorable outcome for bird health and reproductive efficiency.

### External egg quality traits

Although selection primarily targeted production traits, its impact on external egg quality characteristics was also assessed. Selection effects on external egg quality were limited. While shell weight% and shell thickness declined significantly across generations (Table 3), only shell thickness showed significant line-related differences, favoring the SHEW₉₀ line. The shell weight% values remained within normal ranges reported by^29^ and exceeded those reported by^30^ for Czech and Oravka hens. The shell thickness means were lower than those reported by^31^ but remained within the ranges reported by^29,32^.

Shape index values consistently exceeded previous studies reporting ranges of 70.69–76.42%^31^ and 73.66–75.18%^30^ The superior shell thickness in the SHEW₉₀ line agrees with^33^, who reported positive correlations between egg weight and shell thickness in laying hens.

Despite the selection effect for enhanced production traits, all shell quality parameters remained within reference ranges established by previous studies, suggesting balanced adaptation rather than detrimental consequences of genetic improvement, consistent with research by^1^.

### Internal egg quality traits

Internal egg quality traits and yolk cholesterol exhibited generation- and line-specific responses, reflecting underlying physiological and genetic shifts.

Significant differences in yolk weight% were observed between generations, indicating a progressive decrease in relative yolk content over successive generations. Among lines, SHEW₉₀ exhibited significantly lower yolk weight% compared to both SHEN₉₀ and RBCL lines. This reduction reflects that selection for increased egg weight primarily enhanced albumen deposition rather than yolk enlargement, resulting in proportional dilution of yolk percentage despite absolute yolk weight increases. These findings align with^34^, who reported that larger eggs contain proportionally less yolk, regardless of genotype differences or hen age effects.

Significant differences in Haugh units were found between generations, with values ranging from 74.63 to 75.40, suggesting that selection for different production traits did not compromise albumen quality. Our values fall between those reported by^30,32^, indicating maintenance of acceptable albumen quality standards.

Significant differences, without a consistent trend, in yolk index were observed among generations, among lines, SHEN₉₀ demonstrated superior yolk sphericity (45.15). The observed values were generally consistent with the literature, falling within the range of 40.50–46.25.50.25 as documented by^29^. The significant generation × line interaction suggests that selection for higher egg number (SHEN₉₀) may positively influence yolk sphericity and membrane strength, potentially enhancing resilience to storage and handling stresses. The combined improvement in yolk index across generation by line interaction is particularly valuable, as these parameters collectively ensure superior consumer acceptance, enhanced functional properties for food processing applications, improved handling resistance, and extended storage stability of eggs^35^.

A substantial decrease in yolk cholesterol levels was observed from the second generation to subsequent generations in cholesterol content. Significant differences were also found among lines, with the RBCL line showing higher cholesterol content compared to both selected lines. The cholesterol levels in the third and fourth generations were lower than those reported for both Czech hens and Oravka hens by^30^, demonstrating that the current selection program has effectively reduced cholesterol content below typical values. The reduction in yolk cholesterol observed in later generations and selected lines represents a potential market advantage, as low-cholesterol eggs can command premium prices in health-conscious consumer segments^36^.

Generation × line interaction showed significant effects on all studied traits: albumen weight%, yolk weight%, yolk index, Haugh units, and yolk cholesterol, indicating that the selection lines responded differently across generations for these traits.

### Genetic gain and correlated changes

The estimated heritability values provided further insight into genetic architecture and potential for improvement of the studied traits. By dividing the traits into a set of categories, it was observed that serum constituents’ traits were marked with the highest heritability estimates (0.21–0.31), indicating a moderate degree of genetic control, suggesting them as useful candidates for inclusion in a multi-trait selection program. Moreover, the moderate heritability estimates observed for EM traits (0.15–0.21) supporting their responsiveness to selection. Similar heritability estimates for EN₉₀, EW₉₀, and EM₉₀ were observed by^8^. The heritabilities for AFE, EN_90_, EW_90_, and EM_90_ were lower than estimates reported by^33,37^, yet higher than those reported by^38,39^. Notably, the EN_90_ heritability value exceeded that of Thai native chicken selected for EN-45 weeks of age^40^.

Regarding egg quality traits, most of them showed low heritability estimates (0.09–0.16), indicating limited potential for rapid improvement through selection alone. The heritability estimates for shell thickness, shape index, and yolk weight were lower than those found by^33,41^ in Chinese Wenchang and Rhode Island Red chickens. For blood parameters, serum triglyceride heritability in the present study was lower than reported by^42^ (0.31) but higher than values from^43^ (0.02). Conversely, Serum cholesterol heritability in the present study exceeded the estimate of 0.17 reported by^42^. However, these values were consistent with the wider heritability range of 0.14–0.74 previously documented for Alexandria chickens by^44,45^. The current study’s heritability estimates for yolk cholesterol were lower than those reported by^41^. These variations in genetic parameter estimates between studies likely come from differences in populations, estimation methods, statistical models, selection criteria, and breeding program approaches^46^.

The observed genetic gains and correlated trait changes reinforced the relevance of partial-record selection as a practical breeding approach. Direct selection responses were successfully realized in both lines, aligning with moderate heritability estimates, confirming sufficient genetic variability for effective selection. The effectiveness of partial-record selection criteria comes from positive genetic correlations between early and total egg production, combined with reduced generation intervals that maximize genetic gain^9,47^. This approach allows improvement of full record egg production through selection for EN-12weeks of lay, as supported by^48^, who achieved positive genetic gains in egg production traits targeting increased egg number.

An important antagonistic relationship emerged between egg number and egg weight as SHEN_90_ showed reduced EW_90_ as a correlated trait, while the SHEW_90_ exhibited decreased EN_90_ when selected for heavier eggs. This inverse relationship reflects genetic trade-offs widely reported in previous studies^1,8,38^, indicating biological constraints in simultaneously improving both traits due to negative genetic and phenotypic correlations. The observed genetic gains and correlated trait changes reinforced the need for multi-trait selection approaches to manage antagonism between egg number and egg weight, effectively.

Selection also influenced sexual maturity, with egg number selection resulting in earlier maturity in the SHEN_90_ line and delayed maturity in the SHEW_90_ line. The decreased age at first egg in SHEN90 likely contributed to increased egg numbers during the laying period, while delayed maturity in SHEW_90_ was associated with heavier eggs, consistent with findings by^49,50^.

In the current study, both selection criteria significantly improved egg mass during 90- and 365-day periods, with the SHEN_90_ line showing greater gains, indicating that egg number selection appears more effective for egg mass traits. These findings support previous reports that selection for early egg number can result in sustained improvements in overall productivity^8^.

Selection substantially affected metabolic parameters, with both lines showing reduced serum triglycerides. In addition, the SHEN₉₀ line showed a significant reduction in serum cholesterol. These trends align with the observed generation-related decrease in serum triglycerides and cholesterol (Table 2), which suggests favorable lipid metabolism effects regardless of the selection criterion. However, serum cholesterol responses differed, decreasing in the SHEN_90_ but increasing in the SHEW_90_, indicating egg number selection may have more favorable cholesterol metabolism effects.

Finally, egg quality traits differed markedly between lines. Genetic gains in shell traits were modest, and even negative in the SHEN₉₀ line, suggesting a possible antagonistic relationship between egg number and shell traits. These results reflected the results in Table 3, where selection effects on external egg quality were limited. While shell weight% declined significantly across generations (Table 3), only shell thickness showed significant line-related differences, favoring the SHEW₉₀ line.

The SHEN_90_ line showed reductions in shell weight%, shape index, albumen weight%, yolk weight%, and yolk index and yolk cholesterol, while the SHEW_90_ line exhibited improvements, particularly in external egg quality traits. The desirable impact of selection for egg weight resulted in positive estimates of genetic gain in the external egg quality traits. This could be attributed to the positive correlation between the egg weight and the weight of the separate component of the egg, including eggshell^51,52^. The SHEN₉₀ line demonstrated a marked decrease in yolk cholesterol (− 5.32 mg/g), aligning with its lower blood lipid levels, while the SHEW₉₀ line achieved modest improvements in internal quality traits such as albumen weight and yolk index. These results are biologically relevant, as lower yolk cholesterol may enhance the nutritional profile of eggs, a desirable consumer trait.

### Genetic and phenotypic correlations

The correlation structure between selection criteria and associated traits clarified the biological trade-offs and synergies resulting from selection pressure.

The observed correlation patterns revealed meaningful biological relationships that have significant implications for poultry breeding strategies. The positive correlations between EN_90_ and other production traits (EM_90_, EM_365_, EN_365_) emphasizing its value as a core selection trait and indicate that selection for early laying intensity will simultaneously improve overall egg mass production and annual egg production. This trend was supported by^53^, who reported that egg numbers in different laying periods were positively interrelated with strong correlations to total egg production.

The strong negative genetic correlation between EN₉₀ and AFE (− 0.61) supports the role of early onset of lay in enhancing egg number. This result agrees with findings of^37,54^. However, the positive correlation between AFE and EW_90_ indicates that later-maturing hens could produce heavier eggs. This finding is consistent with^37^, who reported a positive correlation between AFE and egg weight in the Rhode Island Red-White strain. Generally, EW₉₀ showed weak or even negative genetic associations with long-term production traits, highlighting the risk of selecting for egg weight alone to improve these traits.

Interestingly, EN₉₀ had moderate positive correlations with serum triglycerides and cholesterol, while EW₉₀ showed higher correlations with serum calcium and yolk cholesterol, still low in magnitude. This suggests that selection for EN₉₀ may indirectly impact metabolic processes, possibly through higher reproductive activity. The positive correlation between EW90 and most internal egg quality traits is consistent with^33^, who reported that the genetic correlations between egg weight and albumen weight, yolk weight, and eggshell weight were positive in brown-egg dwarf layers. Biochemical parameters, particularly serum triglycerides, show meaningful correlations with production traits, indicating potential physiological markers for production. These relationships suggest that metabolic efficiency and lipid metabolism play crucial roles in determining laying performance, offering opportunities for incorporating physiological indicators into breeding programs. All in all, the correlations between selection criteria and egg quality traits were generally weak, reinforcing the observation that selection for egg number or weight is unlikely to produce substantial improvements in quality traits unless directly included in the selection objective. Furthermore, the controlled experimental environment minimized confounding variation, but it also limits broad applicability; future studies should test under diverse field conditions. The metabolic responses reported here represent short- to mid-term effects; long-term selection over additional generations, to capture cumulative and potentially epigenetic effects of sustained selection pressure, will clarify whether sustained physiological adaptation occurs.

In practice, there are logistical and financial obstacles when implementing multi-trait selection in native or smallholder poultry systems. The use of comprehensive selection indices is frequently limited by small flock sizes, inconsistent pedigree recording, and nutritional variability^55,56^. However, while still obtaining detectable genetic gain, partial-record selection for traits like egg number during the first 90 days (EN₉₀) can reduce recording costs and shorten generation intervals^57,58^. It has been demonstrated that adding egg weight and specific physiological markers, like serum cholesterol or calcium, to simplified multi-trait indices produces a balanced increase in egg mass and quality without sacrificing adaptability^59^. This strategy provides a realistic framework for sustainable genetic progress in local breeds like Dokki-4 under resource-limited conditions common to smallholder production systems.

## Conclusion

This study demonstrates that Dokki-4 laying hens’ egg production and metabolic efficiency can be enhanced using partial-record selection. It maintains acceptable physiological profiles and standards for egg quality while increasing productivity. Breeding plans that strike a balance between quality, performance, and metabolic health can be firmly based on the estimated genetic parameters. Crucially, in line with consumer demands and animal welfare, this work promotes sustainable genetic improvement of the local strain and provides insightful information about the biology of egg production. However, antagonistic relationships between egg number and weight highlight the need for balanced multi-trait long-term selection strategies. Overall, partial-record selection offers a practical, cost-effective approach for genetic improvement in local chicken strains, especially under limited-resource breeding environments.

## Data Availability

The data supporting this manuscript’s findings are available upon request from the corresponding author.
